# The Survey of the Health of Wisconsin (SHOW) Program: An Infrastructure for Advancing Population Health

**DOI:** 10.3389/fpubh.2022.818777

**Published:** 2022-03-31

**Authors:** Kristen M. C. Malecki, Maria Nikodemova, Amy A. Schultz, Tamara J. LeCaire, Andrew J. Bersch, Lisa Cadmus-Bertram, Corinne D. Engelman, Erika Hagen, Laura McCulley, Mari Palta, Allison Rodriguez, Ajay K. Sethi, Matt C. Walsh, F. Javier Nieto, Paul E. Peppard

**Affiliations:** ^1^Department of Population Health Sciences, School of Medicine and Public Health, University of Wisconsin, Madison, WI, United States; ^2^School of Medicine and Public Health, Wisconsin Alzheimer's Institute, University of Wisconsin, Madison, WI, United States; ^3^Department of Kinesiology, School of Education, University of Wisconsin, Madison, WI, United States; ^4^College of Public Health and Human Sciences, Oregon State University, Corvallis, OR, United States

**Keywords:** SHOW, population health science, equity, survey, life-course, epidemiology, molecular epidemiology

## Abstract

**Introduction:**

The Survey of the Health of Wisconsin (SHOW) was established in 2008 by the University of Wisconsin (UW) School of Medicine and Public Health (SMPH) with the goals of (1) providing a timely and accurate picture of the health of the state residents; and (2) serving as an agile resource infrastructure for ancillary studies. Today, the SHOW program continues to serve as a unique and vital population health research infrastructure for advancing public health.

**Methods:**

SHOW currently includes 5,846 adult and 980 minor participants recruited between 2008 and 2019 in four primary waves. WAVE I (2008–2013) includes annual statewide representative samples of 3,380 adults ages 21 to 74 years. WAVE II (2014–2016) is a triannual statewide sample of 1,957 adults (age ≥18 years) and 645 children (age 0–17). WAVE III (2017) consists of follow-up of 725 adults from the WAVE I and baseline surveys of 222 children in selected households. WAVEs II and III include stool samples collected as part of an ancillary study in a subset of 784 individuals. WAVE IV consists of 517 adults and 113 children recruited from traditionally under-represented populations in biomedical research including African Americans and Hispanics in Milwaukee, Wisconsin.

**Findings to Date:**

The SHOW resource provides unique spatially granular and timely data to examine the intersectionality of multiple social determinants and population health. SHOW includes a large biorepository and extensive health data collected in a geographically diverse urban and rural population. Over 60 studies have been published covering a broad range of topics including, urban and rural disparities in cardio-metabolic disease and cancer, objective physical activity, sleep, green-space and mental health, transcriptomics, the gut microbiome, antibiotic resistance, air pollution, concentrated animal feeding operations and heavy metal exposures.

**Discussion:**

The SHOW cohort and resource is available for continued follow-up and ancillary studies including longitudinal public health monitoring, translational biomedical research, environmental health, aging, microbiome and COVID-19 research.

## Introduction

Established in 2008 the Survey of the Health of Wisconsin (SHOW) (1) is a one-of-a-kind resource for innovative, cutting-edge population health sciences. Funded by the University of Wisconsin School of Medicine and Public Health endowment funds, SHOW provides a unique resource to address key gaps to advance population health and translational research. Since its inception, the SHOW program has addressed numerous Centers for Disease Control and Prevention (CDC) Public Health 3.0 recommendations for gathering multilevel data on key social determinants of health and engaged multiple stakeholders and community partners to generate collective impact (2).

The widespread and unequitable impacts of the COVID-19 pandemic provide a spotlight on the population health challenges facing both the United States and the global community in the 21st Century. Particularly alarming in the United States was an acceleration of persistent ongoing disparities across and within communities contributing to a 10-fold difference and reduction in life expectancy across the United States. In 2016, the United States CDC called for a new Public Health 3.0 approach to tackle public health challenges in the modern era (2). The new approach calls for multisectoral partnerships necessary to address the social, environmental and economic forces shaping population health. This call to action among public health leaders is analogous to the ongoing movement among population health scientists to identify and address the multiple social determinants of health within health care and community settings.

Recommendations in both population health sciences and public health include the need for more detailed and timely data for public health practitioners, health care providers, community leaders and policy makers. More data on geographical levels are also necessary to support cost effective programming. This more granular data can also be used to better address community-specific social, environment and economic factors driving health and health equity. While several national level surveys exist, sub-population—below state level—data are not often available to describe the unique sub-population differences in social determinants of health and how they change over time and space within and across communities.

Using the entire state population of Wisconsin as a sampling frame, SHOW provides a unique level of granularity to study the health status of individuals and social determinants of health across rural and urban contexts. More recently, focused recruitment efforts have aimed to expand the core study population to include children and increase the racial, ethnic and socio-economic diversity of the study population. Other distinctive elements of SHOW include the geographically diverse study population, the breadth of objective and biological data collected, the ability to link social and environmental contextual data, and the flexibility of the program to support translational science and health equity research.

To date, no other statewide study sample exists. From its inception, SHOW aimed to capture multi-level determinants of data to examine proximate and distal factors shaping health and wellbeing. Detailed data on household address and residential history can be integrated with objective health and biomarker data to advance understanding of how protective and adverse physical and social environments impact biological mechanisms underlying aging trajectories and shape health and wellbeing in a geographically diverse population.

Core funding for SHOW is provided by the Wisconsin Partnership Program and additional ancillary funding from the National Institutes of Health and the Wisconsin Department of Health Services, among others. Scientific direction is provided by experts in population health research from across the entire University of Wisconsin-Madison campus, including a Scientific Advisory Board. Field data collection continues today with numerous opportunities for investigators to inform longitudinal follow-up and clinical collaborations including opportunities for linkage with electronic health records and other administrative data.

## Materials and Methods

### Study Design

SHOW was not originally designed with a specific set of hypotheses in mind but with a broader mission to improve understanding of the multi-level determinants of health and equity, originally emphasizing chronic diseases in adult populations. The core survey contents were therefore determined using a social determinants of health framework. Unique elements of the SHOW design included physical examination combined with interview and bio-specimen collection. Table 1 outlines the breadth of questionnaire, physical exam and biomarker data collected among SHOW participants. Whenever possible, questions were selected from previously validated questionnaires.

**Table 1:** SHOW core components.

	**Topics covered**	
**Primary data collection**		
Self- administered questionnaires—online	•Prevention and safety habits • Diet (Block Screener,^1^ other dietary habits) • Discrimination, adverse child/life events inventory • Smoking and alcohol habits, food security^2^ • Resilience, coping • Food Security (USDA)^3^	•Sleep habits and problems • EuroQol (health-related quality of life)^4, 5^ • Mental health: depression (DASS)^6, 7^PHQ-8^8^ • Self-reported physical activity • Perception on quality of local environment, safety • Access to healthy food, green space, etc.
Computer Assisted Personal Interviews (CAPI)—over the phone, or in person if preferred	•Tracking information • Demographics and occupational history/military • Environmental exposures, housing, pets etc. • Health history, insurance, access & utilization • Prescription and over the counter medications	•SF-12 (health-related quality of life)^9 10^•Cognitive function, health literacy (STOFHLA)^3^ • Residential history • Cancer prevention and control, screening • Consent for EHR, administrative data linkages
Physical exam, biological sample collection blood, urine, DNA, stool	•Weight; height; waist, hip, and arm circumference • Phlebotomy and urine collection • Stool, skin, nasal swab^**^	•Sitting blood pressure and pulse, body fat^11^ • Actigraphy, 7-day-NHANEs protocol^12, 13^ (PA,Sleep) • NCI 24-Hour Dietary recall (online)
**Environmental exposures and response biomarkers**			
Biomarkers for Immediate Research	•Blood – DNA extraction, baseline blood chemistry (CBC with differentials, a lipid panel including total cholesterol, HDL and LDL cholesterol, and triglycerides, glucose, and HbA1c) • Stool - gut microbiome – 16srRNA sequencing, metagenomics • Blood Chemistry	
Biospecimen storage for future research and *examples of potential uses*	•DNA for genetics, epigenetics, telomere and markers of DNA damage and repair • Urine - nitrate, heavy metal exposures • PBMCs cell specific response • RNA for transcriptomics • Whole blood, urine, plasma, serum for future unspecified research • Stool DNA - *metagenomic/deep sequencing for bacteria, fungi and viruses; PCR for specific pathogens* • Plasma/Serum – *untargeted and targeted metabolomic analyses for xenobiotics and functional assessment of metabolic pathways, biomarkers of inflammation*	
**Contextual data**			
GIS-based indicators^*^ of social determinants, health care access, and environmental determinants	•Demographics, economic hardship index • Income, housing and racial inequality^14−16^ • Proximity to health care • Land use	•Traffic use/density; air quality • Density of grocery/convenience stores/fast food • Green space proximity to parks, trails, clinics • Drinking water source, treatment

* *All participants' household addresses are geocoded for linkage with GIS based data including census*.

** *subset WARRIOR*.

Several ancillary study projects have been done in collaboration with community partners who have extracted a smaller number of survey questions important for goals and dissemination. The core infrastructure values community engagement in all aspects of ancillary study development. Thus, the protocols are flexible enough to add new collection tools relevant to study hypotheses as needed (3).

### Study Population

**Figure 1:**
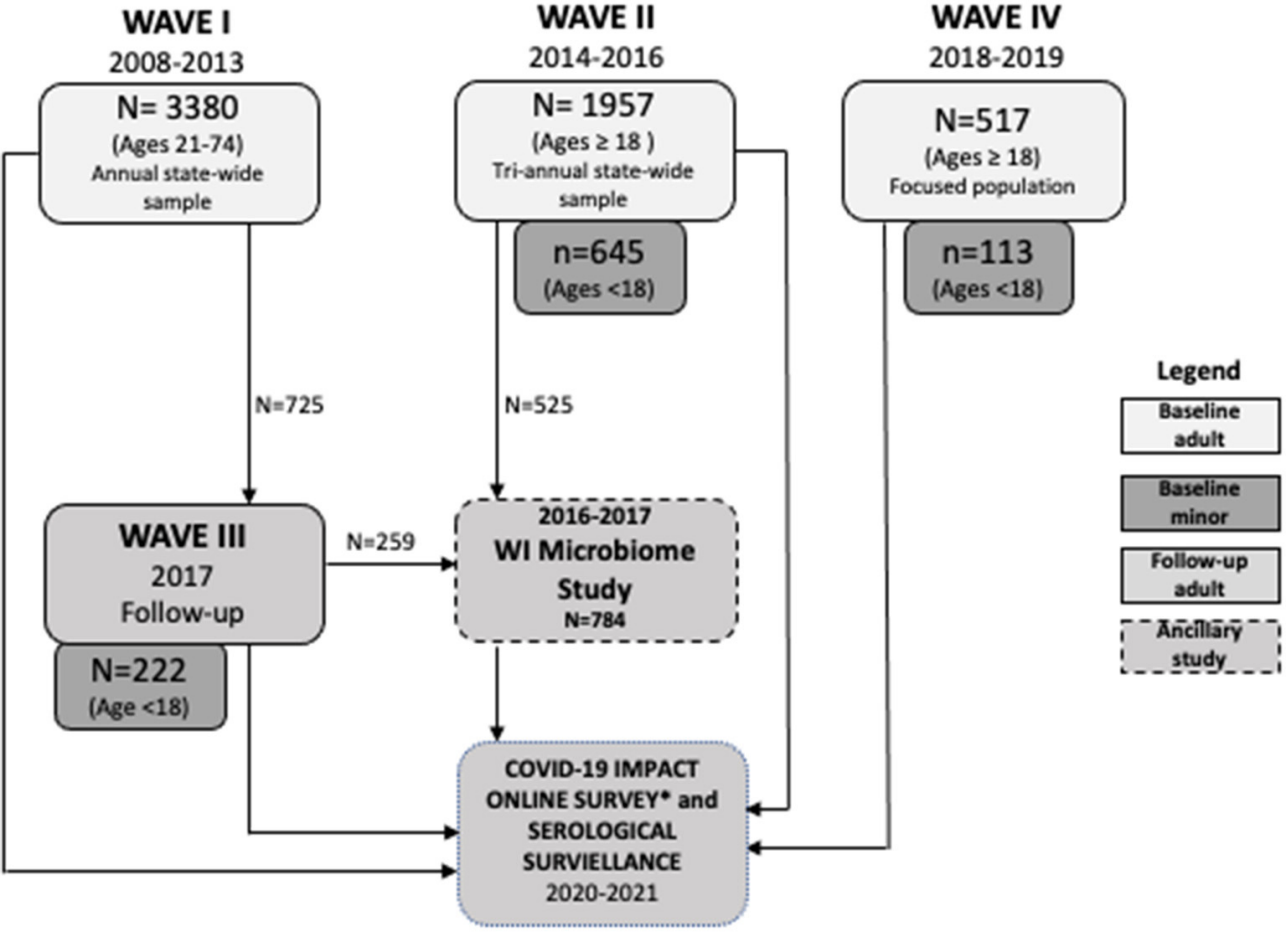
Survey waves and follow-up participation.

Since 2008, the SHOW program has conducted as a series of cross-section and longitudinal surveys on the health status of individuals and social determinants of health. The target study population includes a representative sample of state residents with focused sub-population analyses among largely under-represented populations. The full study sample includes 5,846 adult (ages 18 years and over) and 980 minor (age 0–17 years) participants. Table 2 depicts the multiple waves of data collection and highlights key additions and changes to the cohort composition, sampling strategy, inclusion and exclusion criteria and study components over time. In brief, participants have been recruited across three waves (WAVE 1: 2008–2013, WAVE II: 2014–2016, and WAVE IV: 2018–2019). The first longitudinal follow-up of WAVE I participants was completed in 2017, and is referred to as WAVE III. Figure 1 outlines the recruitment efforts and samples sizes across each study wave. WAVE IV included additional community-engagement and community led recruitment of under-represented Black and Hispanic residents living in Milwaukee, the most highly urbanized community in the state. SHOW protocols aim to provide consistency across each wave of data and biosample collection. All procedures for data collection follow-strict quality assurance and quality control guidelines. Table 3 describes the various recruitment strategies and eligibility criteria across waves.

Diverse state and local partnerships, ongoing community engagement, a detailed website, newsletters and data briefs support recruitment and retention of the cohort. Supplementary Table 1A shows improvement in response rates, measured as number of participants screened eligible willing to participate in the program, over time, by health region and 10 counties that correspond to each health region. Health regions are defined as geographic clusters of counties within a public health service area defined by the Wisconsin Department of Health Services. Supplementary Table 1B Supplementary Table 1B shows response rates by urbanicity as defined by the U.S. Census. Details regarding the design and data collection for each SHOW wave are briefly described below.

#### WAVE I–The Original SHOW Study Sample (2008–2013)

WAVE I (2008–2013) includes a statewide representative sample of 3,380 adults ages 21 to 74 years with key demographics presented in Table 2A. As previously described by Nieto et al. a state-wide address-based sampling frame and two-stage, area probability sampling without replacement (PPSWOR) was used to generate an annual statewide representative sample (1). Selection criteria included age between 21 and 74 years, and residency within the state for longer than 6 months. Exclusion criteria included limited ability to consent independently, active-duty military service, being institutionalized, and undergoing community or home corrections monitoring. The annual sample size ranged from ~300–900 between 2008 and 2013. Response rates ranged from 43 to 87% depending on region across the state and, on average, tended to be higher in rural communities and lower in urban and lower income communities (Supplementary Table 1B). Approximately 80% of participants who completed the household interview went on to complete all survey components (personal in-home interviews, self-administered questionnaire, physical exam, and biosample collection). Survey weights that incorporate design weights and adjustments for non-response and post-stratification, calibrated to the U.S. Census 2010 population totals by age, sex and race, improve the representativeness of statewide estimates, and design variables account for spatial clustering in the sample design.

**Table 2A:** SHOW adults WAVES I and II characteristics, weighted for statewide sample estimation.

	**WAVE I** **2008-2013**			**WAVE II** **2014-2016**		
**Demographic characteristics**	N^*^	Mean or %^*^	Range or 95% CI^*^	N*	Mean or %^*^	Range or 95% CI^*^
**Age (years)**	3,380	45.6	21 - 74	1957	48.7	18 - 98
18 to 29	512	16.6	(14.3, 18.9)	278	15.8	(12.5, 19.1)
30 to 39	592	20.7	(18.4, 23.1)	346	20.7	(17.4, 24.1)
40 to 49	690	21.3	(19.3, 23.3)	255	14.2	(11.4, 16.9)
50 to 59	813	23.1	(21.2, 25.1)	353	19.2	(17.4, 21.0)
60 to 74	773	18.2	(16.5, 20.0)	525	22.5	(18.8, 26.2)
75 or older	NA	NA	NA	200	7.6	(6.0, 9.1)
**Gender**						
Male	1,479	50.1	(48.5, 51.8)	864	49.1	(47.2, 50.9)
Female	1,901	49.9	(48.2, 51.5)	1093	50.9	(49.1, 52.8)
**Race/ethnicity**						
Non-Hispanic white	2,867	85.1	(83.0, 87.3)	1623	85.0	(81.7, 88.2)
Non-Hispanic black	243	6.1	(4.7, 7.6)	151	6.3	(3.6, 9.1)
Hispanic	108	4.1	(2.8, 5.3)	77	3.9	(2.8, 5.0)
Other	154	4.7	(3.3, 6.0)	104	4.8	(3.9, 5.7)
**Education**						
Less than HS	258	7.5	(6.3, 8.7)	132	6.5	(4.9, 8.1)
HS degree or some college	1,416	40.7	(38.1, 43.3)	775	40.1	(37.7, 42.4)
Associate's degree or higher	1,701	51.8	(49.1, 54.4)	1048	53.5	(50.2, 56.7)
**Poverty**						
≤ 200% FPL^+^	985	29.0	(26.4, 31.5)	556	30.5	(26.7, 34.2)
> 200% FPL	2,249	71.0	(68.5, 73.6)	1303	69.5	(65.8, 73.3)
**Employed (among the economic labor force)**					
Yes	2,283	91.1	(89.7, 92.5)	1115	92.6	(90.7, 94.5)
No	238	8.9	(7.5, 10.3)	92	7.4	(5.5, 9.3)
**Health insurance coverage over the last 12 (months)**						
0	316	9.1	(7.7, 10.4)	75	4.1	(2.3, 5.9)
1 to 11	216	6.3	(5.3, 7.3)	146	8.3	(7.0, 9.5)
12	2,833	84.6	(82.9, 86.4)	1742	87.6	(84.7, 90.5)
**Census 2010 urban / rural classification**						
Urban	2,139	67.1	(61.4, 72.7)	1339	69.9	(48.8, 90.9)
Rural	1,241	32.9	(27.3, 38.6)	618	30.1	(9.1, 51.2)

* *Unweighted. ^+^FPL, federal poverty level; HS, high school*.

** *Weighted and adjusted for the stratification and clustering in the complex survey sampling design*.

*Frequencies may not add to the total sample size due to missing values*.

#### WAVE II–SHOW Tri-Annual Expansion (2014–2016)

WAVE II, SHOW 2014-2016, provided a newly recruited prospective tri-annual statewide representative sample of 1,957 adults (age ≥18 years) and 645 children (<18 years of age). Demographic data for the adult sample are presented in Table 2B while children are presented in Table 2C. Eligibility criteria for WAVE II expanded to add children (<18). Adult participants of any age, with ability to individually consent without cognitive or other impairments were also included. All other exclusion criteria were consistent with WAVE I. Similar to WAVE I, an area probability sampling design was used to randomly select households, where all eligible household members were invited to participate. Unlike WAVE I, the two-stage sampling design was modified to three-stages with county as the primary sampling unit (PSU) rather than Census block group (CBG) in WAVE II. The statewide representative sample became a tri-annual rather than annual sample. Eight PSUs, stratified by years of potential life lost, were randomly selected with probabilities proportional to size where the measure of size was occupied housing units. Two counties (Milwaukee and Dane) were selected with certainty (probability of selection = 1) based on their large number of occupied housing units relative to the other counties. CBGs served as secondary sampling units with poverty stratification, and households within each CBG were randomly selected using simple random sampling.

**Table 2B:** SHOW adults WAVES III and IV characteristics, unweighted.

	**WAVE III** **Follow-up 2017**			**WAVE IV** **Focused population oversample**		
**Demographic characteristics**	**N**	**Mean or %**	**Range or 95% CI**	**N**	**Mean or %**	**Range or 95% CI**
**Age (years)**	725	54.1	25 - 82	517	46.8	18 - 91
18–29	29	4.0	(2.6, 5.4)	92	17.8	(14.5, 21.1)
30–39	114	15.7	(13.1, 18.4)	94	18.2	(14.8, 21.5)
40–49	128	17.7	(14.9, 20.4)	94	18.2	(14.8, 21.5)
50–59	157	21.7	(18.6, 24.7)	110	21.3	(17.7, 24.8)
60–74	238	32.8	(29.4, 36.3)	111	21.5	(17.9, 25.0)
75 or older	59	8.1	(6.1, 10.1)	16	3.1	(1.6, 4.6)
**Gender**						
Male	288	39.7	(36.2, 43.3)	199	38.5	(34.3, 42.7)
Female	437	60.3	(56.7, 63.8)	318	61.5	(57.3, 65.7)
**Race/ethnicity**						
Non-Hispanic white	575	79.5	(76.6, 82.5)	33	6.4	(4.3, 8.5)
Non-Hispanic black	96	13.3	(10.8, 15.8)	339	65.6	(61.5, 69.7)
Hispanic	22	3.0	(1.8, 4.3)	125	24.2	(20.5, 27.9)
Other	30	4.1	(2.7, 5.6)	20	3.9	(2.2, 5.5)
**Education**						
Less than HS	47	6.5	(4.7, 8.3)	159	30.8	(26.8, 34.7)
HS degree or some college	272	37.5	(34.0, 41.0)	249	48.2	(43.8, 52.5)
Associate's degree or higher	406	56.0	(52.4, 59.6)	109	21.1	(17.6, 24.6)
**Poverty**						
≤ 200%^+^ FPL	167	23.7	(20.5, 26.8)	344	74.9	(71.0, 78.9)
> 200% FPL	539	76.3	(72.2, 79.5)	115	25.1	(21.1, 29.0)
**Employed (among the economic labor force)**						
Yes	450	95.3	(93.4, 97.2)	220	72.6	(67.6, 77.7)
No	22	4.7	(2.8, 6.6)	83	28.4	(22.3, 32.4)
**Health insurance coverage over the last 12 months**						
0 (months)	12	1.7	(0.7, 2.6)	55	12.3	(9.3, 15.4)
1–11 (months)	30	4.1	(2.7, 5.6)	50	11.2	(8.3, 14.2)
12 (months)	681	94.2	(92.5, 95.9)	341	76.5	(72.5, 80.4)
**Census 2010 urban/rural classification**						
Urban	575	79.3	(76.4, 82.3)	517	100.0	NA
Rural	150	20.7	(17.7, 23.6)	NA	NA	NA

*Frequencies may not add to the total sample size due to missing values. ^+^FPL, federal poverty level; HS, high school*.

**Table 2C:** SHOW children in WAVES II, III and IV characteristics.

	**WAVE II** **2014-2016**			**WAVE III** **2017**			**WAVE IV** **2018-2019**		
**Demographic characteristics**	N*	Mean or %^**^	Range or 95% CI^**^	N*	Mean or %^*^	Range or 95% CI^*^	N^*^	Mean or %^*^	Range or 95% CI^*^
**Age (years)**	645	7.7	0 - 17	222	8.6	0–17	113	8.0	0–17
0–6	279	44.8	(39.4, 50.2)	71	32.0	(25.8, 38.2)	49	43.4	(34.1, 52.6)
7–11	182	28.1	(25.5, 30.8)	88	39.6	(33.2, 46.1)	27	23.9	(15.9, 31.9)
12–17	184	27.1	(22.2, 31.9)	63	28.4	(22.4, 34.4)	37	32.7	(24.0, 41.5)
**Gender**									
Male	332	51.1	(46.5, 55.8)	123	55.4	(48.8, 62.0)	59	52.2	(42.9, 61.6)
Female	313	48.9	(44.2, 53.5)	99	44.6	(38.0, 51.2)	54	47.8	(38.4, 57.2)
**Race/ethnicity**									
Non-Hispanic white	472	71.4	(62.9, 79.8)	149	67.4	(61.2, 73.6)	4	3.6	(0.1, 7.1)
Non-Hispanic black	103	16.8	(8.2, 25.5)	38	17.2	(12.2, 22.2)	101	90.2	(84.6, 95.8)
Hispanic	15	2.5	(0.2, 4.7)	23	10.4	(6.3, 14.5)	6	5.4	(1.1, 9.6)
Other	53	9.3	(5.5, 13.2)	11	5.0	(2.1, 7.9)	1	0.9	(0.0, 2.7)

* *Unweighted*.

** *Weighted and adjusted for the stratification and clustering in the complex survey sampling design*.

*Frequencies may not add to the total sample size due to missing values*.

Response rates were slightly higher on average in WAVE II with 64% of screened eligible individuals agreeing to participate (see Supplementary Table 1B). This higher response rate was attributed to additional focus on identifying field interviewers representative of the targeted community, and additional focus on community engagement and awareness campaigns, including endorsement by local officials prior to recruitment. Finally, we aimed to improve the ease of exam visits and sample collection by identifying exam visit locations in places of worship, or other locally respected locations that were convenient and centrally located for study participants. Design variables that account for clustering in the sampling design and survey weights based on design weights adjusted for non-response and calibrated to the U.S. Census Current Population Survey 2016 estimates by age, sex and race are available for WAVE II.

#### WAVE III–Follow-Up for Wave I Participants

WAVE III included longitudinal follow-up of n=725 adults from WAVE I (see Table 2B) and baseline participation of 222 children (see Table 2C). The eligibility criteria for WAVE III were participation in WAVE I, consent to be contacted by SHOW for future studies, WAVE I residents in 13 select counties cover the full spectrum of urbanicity and county health rankings across Wisconsin. For Non-Hispanic white participants, additional eligibility criteria were completion of the physical examination and biomarker collection in Wave I. All children currently residing in follow-up participant households were also eligible.

WAVE III follow-up included an in-home interview, physical exam, core biospecimen collection (blood, urine) and stool and skin swabs collection for microbiome analysis funded via ancillary study funding described below. Follow-up participation rate, determined based on number of those contacted who agreed to participate again, was estimated at 86% (see Table 3). Survey weights were not generated for WAVE III since it was not a random subsample of WAVE I.

**Table 3:** SHOW survey participant summary, sampling strategy and components by WAVE.

	**WAVE I**	**WAVE II**	**WAVE III**	**WAVE IV**
	**Baseline**	**Baseline**	**Follow up**	**Baseline**
Timeline	2008–2013	2014–2016	2017	2018–2019
Number of participants enrolled	Adults: 3,380	Adults: 1,957; Minors: 645	Adults: 725; Minors: 222	Adults: 517; Minors: 113
Sampling strategy	Annual state-wide representative samples	Tri-annual state-wide representative sample	Wave I participants	Focused recruitment among African Americans and Hispanics
Response rate	57.5%	63.5%	85.6%	NA
Eligibility criteria	Age 21–74; WI* resident for at least 6 months	All ages; WI resident for at least 6 months	Participation in Wave I; minors living in participants households	All ages; WI resident for at least 6 months
Exclusion criteria		Active duty military service
		Being institutionalized
		Undergoing correction monitoring
		Limited ability to consent independently
Survey components		CAPI (Computer Assisted Personal Interview)
		physical measurements
		SAQ (Self-Administered Questionnaire)
		Biosample collection

*WI, Wisconsin*.

#### WAVE IV—Focused Recruitment of Traditionally Under-Represented Populations in Biomedical Research

In 2018–2019 SHOW focused on engaging and recruiting participants from two traditionally under-represented populations in biomedical research including an oversample of 440 Black (339 adult and 101 minor) and 131 Hispanic (125 adult and six minor) participants living in and around the City of Milwaukee (see Table 2B for demographic details on adults and for Table 2C minors). Unlike in WAVES I and II, both two-stage area probability sampling and community engaged convenience sampling approaches using community-based events were employed as primary recruitment strategies. The two-stage area probability sampling design was analogous to WAVE I, with the exception that the PSU sampling frame was restricted to 236 CBGs in the City of Milwaukee with populations of at least 60% African Americans based on the American Community Survey from 2015.

Alternative convenience-based recruitment strategies were developed collaboratively with and in response to community partners' interests in using an asset-based, community-driven model to guide research in the City of Milwaukee. Collaborations were led by investigators with the University of Wisconsin Center for Community Engagement and Health Partnerships (CCEHP) (4). The partnerships and stakeholders informed all aspects of recruitment, including promotion opportunities, use of community events and modifications to survey content relevant to stakeholder interests. Survey elements were modified for use in Hispanic populations and Spanish translation of the final survey content approved by CCEHP partner organizations. Survey weights are not available for WAVE IV due to the hybrid nature of the sampling approach.

### Interviews and Questionnaires

The in-home visit by field interviewers includes computer-assisted personal interviews (CAPI) to gather information on health history and important covariates such as occupation, home environment, health care access, medication use, and demographics (1). Several self-administered questionnaires either on paper or increasingly offered online are used to gather detailed information capturing a broad array of social determinants including food security and economic hardship, personal and family medical history, mental health and wellbeing, quality of life, every day and lifetime racial and other discrimination, life evets, resilience, and coping scales. A neighborhood perceptions questionnaire captures community assets and perceived neighborhood stressors. A personal exposure history (5–7), includes information on residential history, household characteristics including the age of the home, pet ownership, use of indoor/outdoor pesticides, and smoking policies and water source (private well vs. municipal) (8) including use of water filtration. Health behaviors include physical activity, diet, sleep, smoking, and drug and alcohol use. Diet information are captured using both the NCI food frequency questionnaire (all WAVES) and the 24-h dietary recall.

### Physical and Clinical Measurements

In addition to survey data, participants undergo a brief physical exam that includes standardized measurements of blood pressure, weight, height, waist and hip circumference, respiratory function, and collection of blood and urine samples. Weight is measured in kilograms (to a precision of ±0.1 kg) using digital scales with subjects wearing light clothing or surgical scrubs. Height, hip and waist circumference (all in cm) are measured twice. Sitting blood pressure and heart rate are measured using digital blood monitors with three measurements taken 1 min apart after an initial 5-min rest period. Lung function is assessed by spirometry using a Jaeger AM1+ electronic peak flow meter with filter mouthpiece. Testing provides data on FEV1 (forced expiratory volume in 1 s) and FVC (forced vital capacity).

### Wearable Measurement of Objective Physical Activity and Sleep Measurements

Objective physical activity and sleep data are obtained using wearable technology. A detailed protocol for participant 7-day hip and wrist protocol using ActiGraph wGT3X-BT accelerometers (ActiGraph, Pensacola, FL) was developed for both adults, and children >6 years. Data are processed and analyzed using ActiLife software. Both raw and processed data are made available to investigators.

### Biospecimen Collection and Biobanking

All participants providing biological samples are also asked to consent for use of these biological samples for DNA analyses and other future unspecified research. Biological samples, including plasma, serum, urine, DNA, and stool are stored in SHOW's 11 freezers managed by Freezerworks. The growing biobank includes over 200,000 cryovials of urine, plasma, serum, PaxGene and DNA samples stored at −80C for future unspecified research. Following an in-home visit, biological samples are collected either in participant homes or at local exam centers. Several tubes of venous blood (about 55–60 ml in total) are collected and immediately processed for serum and plasma, aliquoted into cryovials and frozen at −80C. A blood aliquot is sent to Marshfield Labs (Marshfield, WI) for complete blood cell count with differential, hematocrit, hemoglobin, HbA1c, glucose, creatinine, triglycerides, total and HDL cholesterol. Blood samples are sent to Prevention Genetics (Marshfield, WI) for DNA extraction. Urine samples are centrifuged, aliquoted into cryovials and frozen.

Starting in 2014, PAXgene tubes for RNA extraction were added to the collection protocol and stool microbiome collection began in 2016. Stool specimens are self-collected using a commercial “toilet hat” collection kit within 12 h of the exam visit. Our current studies have over 95% adherence to this self-collection protocol, including shipping specimens in the correct containers and temperature. DNA from a subset of n=650 participants were analyzed by the NIH Center for Inherited Disease Research (CIDR). The program provided genome-wide MEGA chip array data for identification of SNP polymorphisms, and DNA methylation for epigenetic analyses. The same subset of 650 individuals also have stool microbiome data available.

### Data Analysis

All methods are well-documented through meta-data and online codebooks to ensure rigor and reproducibility over time. Statistical analyses included here include descriptive cross-sectional findings. Use of different statistical methods and approaches are applied as appropriate depending on study aims and research goals. Standard analytic approaches applied to SHOW data include correlation, ANOVA, and multivariate linear and logistic regression analyses. Longitudinal generalized linear models and spline regression have also been employed.

### Ethics

The core SHOW study is approved by the University of Wisconsin Health Sciences Institutional Review Board and all biosecurity and institutional safety procedures are HIPAA compliant. All data and specimens collected on human subjects in the Survey of the Health of Wisconsin are protected from technical and physical loss or damage and from disclosure of identifiable data from the initial point of collection through interim storage, transport, transmissions, downloads, processing, final storage, and distribution of datasets and specimens. All ancillary studies and data requests are also required to obtain appropriate IRB approval prior to data release.

### Consent

Trained field interviewers review consent documents and checklists to assure that participants are informed of all aspects of survey participation prior to consent. Participants may choose to not answer any questions and that they are not required to complete all SHOW components. Incentives for the participation in the program are offered and vary by completion of each survey component. Anonymous feedback forms with self-addressed stamped envelopes are provided to participants following completion of the survey. The longitudinal nature of SHOW also allows for tracking trends over time in the population. Participants are allowed to opt out of data sharing for future unspecified research and can opt out of any future participation. The majority (>90%) of past participants consent for data and biological samples (urine, blood and DNA) to be used for future unspecified research. SHOW has also obtained an NIH Certificate of Confidentiality, to further ensure data will not be shared for reasons outside the original scope of the survey.

### Data Linkages

All participants are geocoded to the household address level that can link to social and environmental data at multiple geographic scales. In addition, all participants are consented for linkage with administrative databases including vital statistics and state cancer registry data. Ongoing efforts are being made to reconsent participants for linkage with electronic medical records and for deposition of genetic and epigenetic analyses into NIH dbGaP database. Socio-demographic and environmental measures can be linked to the data using a street address or other geography indicators (e.g., CBG).

## Key Findings to Date

The breadth and nature of data collected by the SHOW program allows for multidisciplinary research on social determinants of health and numerous outcomes. Focus areas to date include food security, health care access, diet, physical activity, alcohol and drug consumption, prevention behaviors, economics, the built environment including urban and rural exposures, and social and community capital. Outcomes relate to aging, chronic disease, mental health, and other health determinants. They include markers of cardio-metabolic disease (HbA1c, lung function, microbiome), cancer, stress, anxiety, depression and PTSD, cancer prevention and control (9–19). Tables 4A,B describe key findings on health status for WAVES I and II and WAVES II and IV respectively, Supplementary Table 2 highlights the distribution of questionnaires by survey wave. The complete list of over 60 publications is available at www.med.wisc.edu/show. A summary of key findings including those related to COVID-19 follow.

**Table 4A:** Select health indicators for SHOW adults WAVES I and II, weighted for statewide sample estimation.

	**WAVE I 2008–2013**			**WAVE II 2014–2016**		
**Select Health Indicators**	N^*^	Mean or %^**^	95% CI^**^	N^*^	Mean or %^**^	95% CI^**^
**Body Mass Index (kg/m** ^**2**^ **), mean**	2,930	29.5	(29.1, 29.9)	1,914	29.7	(29.1, 30.3)
Underweight (<18.5)	36	1.2	(0.8, 1.7)	21	1.1	(0.5, 1.7)
Normal weight (18.5 to 24.9)	780	26.5	(24.2, 28.9)	497	26.3	(23.5, 29.0)
Overweight (25.0 to 29.9)	935	33.2	(30.7, 35.6)	609	31.6	(28.7, 34.4)
Obese (≥ 30)	1,179	39.1	(36.5, 41.6)	787	41.1	(37.7, 44.5)
**Hemoglobin A1c (%), mean**	2,563	5.7	(5.6, 5.7)	1,376	5.5	(5.4, 5.5)
**Hypertension** ≥ 140/90 mmHg or medication use	303	42.4	(38.8, 46.1)	237	48.6	(44.1, 53.0)
Awareness	223	73.6	(68.6, 78.6)	207	87.3	(83.1, 91.6)
Treatment with medication (among aware)	200	89.7	(85.7, 93.7)	188	90.8	(86.9, 94.8)
Control, <140/90 (among treated)	113	56.5	(49.6, 63.4)	112	59.6	(52.5, 66.7)
Lung function (FEV1/FVC)^***^, mean	2,351	0.84	(0.83, 0.84)	1,642	0.82	(0.79, 0.85)
0.80 to 1.00	1,804	78.3	(75.7, 80.8)	1,167	70.3	(61.3, 79.2)
<0.80	658	21.7	(19.2, 24.3)	475	29.7	(20.8, 38.7)
**Smoking Status**						
Current	555	18.1	(16.3, 20.0)	231	14.3	(11.7, 16.8)
Former	825	27.3	(25.4, 29.2)	485	25.9	(22.0, 29.9)
Never	1535	54.6	(52.3, 56.8)	1010	59.8	(54.7, 64.9)
Depression Severity^***^						
> Moderate to Severe (%)	337	11.9	(10.1, 13.7)	202	12.5	(10.6, 14.4)
Anxiety Severity^***^						
> Moderate to Severe (%)	280	9.59	(7.9, 11.2)	201	12.2	(10.1, 14.3)
Stress Severity^***^						
> Moderate to Severe (%)	159	5.1	(3.9, 6.2)	118	7.6	(6.1, 9.2)
**Food insecurity concern in the last 12 months**	352	12.3	(10.5, 14.2)	275	15.1	(12.3, 17.9)
**Lifetime discrimination instances**						
0	1,319	45.0	(42.3, 47.6)	801	45.7	(40.9, 50.5)
1 or 2	1,010	34.2	(31.9, 36.6)	549	31.0	(27.4, 34.5)
3 or more	628	20.8	(18.6, 22.9)	389	23.3	(21.1, 25.6)
**Neighborhood safe from crime**						
Not very safe or not at all safe	84	2.7	(2.1, 3.3)	90	5.3	(3.4, 7.2)

* *Unweighted*.

** *Weighted and adjusted for the stratification and clustering in the complex survey sampling design*.

*Frequencies may not add to the total sample size due to missing values*.

*** *From the 21 item depression, anxiety and stress scale (DASS21); FEV1, forced expiration volume in one second; FVC1, forced vItal capacity in one second*.

**Table 4B:** Select health indicators for SHOW adults WAVES III and IV, unweighted.

	**WAVE III 2017**			**WAVE IV 2018–2019**		
**Select Health Indicators**	N*	Mean or %*	95% CI**	N*	Mean or %**	95% CI**
**Body Mass Index (kg/m** ^**2**^ **), mean**	716	30.9	(30.4, 31.5)	501	32.1	(31.4, 32.8)
Underweight (<18.5)	6	0.8	(0.2, 1.5)	6	1.2	(0.2, 2.2)
Normal weight (18.5 to 24.9)	156	21.8	(18.8, 24.8)	77	15.4	(12.2, 18.5)
Overweight (25.0 to 29.9)	204	28.5	(25.2, 31.8)	139	27.7	(23.8, 31.7)
Obese (≥ 30)	350	48.9	(45.2, 52.6)	279	55.7	(51.3, 60.1)
**Hemoglobin A1c (%), mean**	508	5.7	(5.6, 5.8)	343	6.1	(6.0, 6.3)
**Hypertension** ≥ 140/90 mmHg or medication use	303	42.4	(38.8, 46.1)	237	48.6	(44.1, 53.0)
Awareness	223	73.6	(68.6, 78.6)	207	87.3	(83.1, 91.6)
Treatment with medication (among aware)	200	89.7	(85.7, 93.7)	188	90.8	(86.9, 94.8)
Control, <140/90 (among treated)	113	56.5	(49.6, 63.4)	112	59.6	(52.5, 66.7)
Lung function (FEV1/FVC)^***^, mean	652	0.84	(0.83, 0.85)	292	0.81	(0.79, 0.82)
0.80 to 1.00	524	80.4	(77.3, 83.4)	227	61.5	(56.5, 66.5)
<0.80	128	19.6	(16.6, 22.7)	142	38.5	(33.5, 43.7)
**Smoking Status**						
Current	79	12.7	(10.1, 15.4)	102	28.7	(24.0, 33.5)
Former	203	32.7	(29.0, 36.4)	58	16.3	(12.5, 20.2)
Never	338	54.5	(50.6, 58.4)	195	54.9	(49.7, 60.1)
Depression Severity^***^						
> Moderate to Severe (%)	78	12.6	(10.1, 15.3)	93	26.9	(22.2, 31.6)
Anxiety Severity^***^						
> Moderate to Severe (%)	67	10.8	(8.3, 13.3)	201	12.2	(10.1, 14.3)
Stress Severity^***^						
> Moderate to Severe (%)	45	7.2	(5.2, 9.3)	118	7.6	(6.1, 9.2)
**Food insecurity concern in the last 12 months**						
Yes (%)	84	11.7	(9.3, 14.0)	146	30.2	(26.1, 34.3)
**Lifetime discrimination instances**						
0	287	45.7	(41.7, 49.5)	88	26.0	(21.3, 30.6)
1 or 2	194	30.8	(27.2, 34.5)	101	29.8	(24.9, 34.7)
3 or more	148	23.5	(20.2, 26.9)	150	44.2	(38.9, 49.6)
**Neighborhood safe from crime**						
Not very safe or not at all safe (%)	39	6.2	(4.3, 8.1)	141	38.3	(33.3, 43.3)

*Frequencies may not add to the total sample size due to missing values*.

*** *From the 21 item depression, anxiety and stress scale (DASS21); FEV1, forced expiration volume in one second; FVC1, forced vItal capacity in one second*.

### Environmental Health

The diverse urban and rural study sample facilitates novel environmental studies examining how psychosocial and physical environments intersect and determine population vulnerability and susceptibility to exposures (14, 16). Objective and subjective measures of physical activity and the built environment continue to support novel methods for behavioral and built environment research in both child and adult populations (20–23).

SHOW was among the first to examine associations between green space and mental health, now a growing area of research (9). We found that a positive neighborhood perception and green space correlate with better sleep quality (24, 25). Moreover, exposure to chronic low-level air pollution has shown adverse associations with lung function and respiratory allergies, which also vary by perception of neighborhood safety and aesthetics (14, 16). Similarly, residential proximity to large dairy concentrated animal feeding operations was also associated with reduced lung function in adults and children (9, 25). Studies using SHOW data have also found populations vulnerable to drinking water contaminants due to limited testing and private well stewardship (8). A follow-up survey of private well-owners in rural communities found limited knowledge and resources to be barriers to well testing, an evidence-based strategy for id**e**ntifying adverse environmental exposures in drinking water (8).

### Health Equity

SHOW also supports comprehensive assessment of multi-level determinants of health and health equity. Common determinants of inequalities are associated with perceived neighborhood safety and aesthetics, access to healthy food and the food retail environment, health care access, oral health and experiences of discrimination (10, 13, 14). Food insecurity has been shown to be prevalent across the entire study population associated with economic hardship in both urban and rural communities and has been associated with several adverse metabolic and cardiovascular outcomes (15, 26). In addition to the statewide representative sample, the SHOW program has made a concerted effort to engage with and recruit from populations traditionally under-represented in biomedical research. In 2018–2019, SHOW conducted focused recruitment to increase the number of African American and Latino participants (27). Compared to the SHOW statewide cancer survivorship prevalence of 12%, the 2018–2019 sample of largely African Americans had a lower prevalence of cancer survivorship, around 9%. At the same time, this group was younger and more likely than the statewide representative sample to identify themselves as a current or former smoker (53% compared to 45% statewide). While further analysis is needed, these trends highlight important trends and sub-population analyses can support future health equity research. A primary goal of the SHOW program is to support health equity using an asset based vs. deficit lense (4), suggesting that within traditionally under-served and marginalized communities, there are tremendous strengths, building on strengths while reducing structural barriers identified in SHOW is a key to advancing health equity.

### Cardio-Metabolic Health and Cancer Research

Objective measures of obesity indicate that over 70% of the state population is overweight or obese, and that a higher level of obesity is correlated with multiple co-morbidities (19). Numerous studies examine predictors of obesity, and determinants of metabolic syndrome in the SHOW population (11, 13, 15, 19, 22, 28). Obesity has also been shown to modify associations of respiratory outcomes with air pollution and smoking exposure in the study sample, suggesting SHOW is a valuable resource for examining the role of obesity in increasing human susceptibility to environmental exposures and the biological mechanisms underlying these associations. Cancer prevention is also a key state health priority with significant disparities. SHOW data have been used by to examine cancer risk factors and policies toward cancer prevention and control including awareness and adherence of radon and private well testing in homes (8, 29), physical activity in both children and adults (21, 23, 30).

### Multi-Omics Research

SHOW's biorespository facilitates research on biological effects of multiple social determinants of health and interim or novel biomarkers of response. Whole blood has been used to examine influences of caregiver strain on telomere length (31–33) and ongoing investigations are examining residential disadvantage on accelerated biological aging and DNA methylation. Analysis of whole blood mRNA levels revealed differential gene expression in stress and toxicity pathways in obese smokers compared to non-obese smokers (34). Plasma, serum, microbiome and mRNA data can also support future metabolomic, lipodomic and transcriptomic research in this well-characterized sample. All of this can support novel exposomic research related to numerous outcomes and phenotypes.

### Evidence for Program Planning, Health Policy, and Translational Research

The program also offers opportunities for both informing health policy and measuring the impact of natural experiments related to significant policy changes (12). SHOW data on use of opioids, and children's screen time have appeared in state policy briefings used to advocate for more comprehensive programs. SHOW surveys have also been used to inform community-driven health assessments, (35) to implement healthy eating interventions (36, 37), and to objectively assess the social and built environment (22), Great Lakes fish consumption, and oral health equity (38–40). Finally, SHOW surveys have informed statewide health guidelines. The Wisconsin Cancer Collaborative conducted a mail-based survey to past SHOW participants identifying as cancer survivors (N = 306). The findings from this study informed Wisconsin's Comprehensive Cancer Control Plan for 2020–2030 including prioritization of patient, provider and caregiver awareness of cancer risk reduction behaviors and screenings for cancer survivors (41).

### Ancillary Studies

Since its inception, numerous ancillary studies have either extended the focus of the baseline SHOW program or facilitated follow-up with cohort participants around particular etiologic, prevention or intervention research questions. Multi-disciplinary research teams and community partners have amplified SHOW's impact over the years through diverse ancillary studies. Examples include personalized vitamin D supplementation based on genetic analysis (42), impacts of caregiver strain on telomere length and quality of life (31–33), assessment of physical activity in rural women (23, 30), and incontinence research in older women (43). Other ancillary studies have examined how the household context impacts personal health information management (44–46), analyzed chronic stress and cardio-metabolic risk (14, 47), and found epigenetic signatures of aging and health disparities, among others. SHOW also supports applied public health and surveillance at the state and local level. Examples of projects with the Wisconsin Department of Health Services include oral health screening (18, 38), as well as a long-standing collaboration to examine the health impacts of Great Lakes fish consumption across the state, among anglers and in high-risk populations (e.g., Burmese immigrants) (39, 40, 48–51).

By tapping into an existing infrastructure, investigators can save time and money and accelerate translational research by supporting multi-disciplinary collaborations. For example, basic science researchers examined branched chain amino acids in 788 human plasma samples (52). Using existing SHOW nutrition, BMI and biosample data, what is typically a costly 5 year study was conducted in 6 months (52). Investigators may use sub-samples of data for new biomarker discovery, comparing biomarker levels from disease free SHOW participants (controls) to clinical patients (cases). Similarly, analyses can examine impacts of exposure among subsets of exposed and non-exposed. For example, transcriptional profiling in a sub-sample of 180 smokers and non-smokers with objectively measured BMI found differential expression of toxic and stress related genes in obese vs. non-obese smokers (34). These translational findings highlight how obesity itself may alter gene expression, increasing vulnerability to environmental threat and findings have implications for therapeutic treatments.

Ancillary studies using the SHOW infrastructure aid basic scientists, clinicians, public health professionals and community leaders in advancing population health in Wisconsin and beyond. For example, clinical investigators used the ongoing collection and follow-up mailings of past participants to examine preferences for receiving information and education on urinary incontinence among women (43). Findings were used to design a follow-up feasibility study needed to inform future implementation research.

### Wisconsin Microbiome Study and Related Resources

Ancillary study funding supported expansion of biological sample collection to include stool, nasal, and skin swabs for microbiome analyses. The Wisconsin Microbiome Study was launched in 2016 to investigate the presence of multi-drug resistant organisms (MDROs) and to characterize the human microbiome in the population (53). SHOW added questionnaires on risk factors for MDRO colonization, diet history, and food-frequency. Stool and swab samples (skin, nasal, oral) were collected from 700 participants and analyzed for MDRO colonization; 16s rRNA gene sequencing data are available for all stool samples collected from this project (53).

In 2018, a subset (59%) of Wisconsin Microbiome Study participants were invited to complete a follow-up visit. Stool and environmental samples (high-touch surface swab, household dust, and soil samples) were collected and are available for future analyses (54). Additional NIH research funded by the National Institutes of Aging and The National Institute of Allergy and Infectious diseases are ongoing. The Wisconsin Microbiome Ancillary Study in children and adults demonstrated the role of xenobiotics and other settings in shaping the human gut microbiome and increased risk for MDRO colonization (53, 55, 56). This represents an important and novel area for metabolic, aging and population health research.

### COVID-19 Impacts on Population Health

As the COVID-19 pandemic emerged in the United States, SHOW shifted efforts toward two specific research efforts which are described in more detail elsewhere (57, 58). In brief, the SHOW program partnered with Wisconsin Department of Health Services and the Wisconsin State Laboratory of Hygiene to conduct antibody surveillance among WAVE II participants (57, 58). The SHOW program also conducted online surveys of COVID-19 impacts on health and well-being over time (May-June, 2020; January-February, 2021; and May-June 2021) among all past SHOW participants. Unique data on subpopulation differences in antibody prevalence and vaccine hesitency were detected as part of the antibody surveillance efforts. Data from the online COVID-19 impact survey highlight the role that exisitng social determiants, including access to care, disabilities, and community capital, played in shaping disparities in COVID-19 testing and adverse economic consequences (57). This important research effort has also allowed SHOW scientists to gather critical information for continuing longitudinal follow-up of the SHOW cohort.

## Discussion

SHOW is a one-of-a-kind resource and infrastructure for accelerating population health science research that has made a tremendous impact in advancing health and health equity in Wisconsin and beyond. Over one hundred peer review or other policy briefs and publications have emanated from the project. Peer-review publications range from basic descriptions of key health determinants (e.g., green-space, obesity, food security) across diverse communities (e.g., urban, suburban and rural), to identification and analyses of complex and previously understudied social determinants (e.g., industrial cow farming). Policy makers have also used data for a variety of policy briefs, including data to support screen time and mental health in children, reduce physical activity barriers for rural women, and advancing cancer prevention and control. Finally, as the resource continues to grow, several local health agencies have partnered with SHOW to identify unique data elements and fill important data gaps for more detailed and robust community health needs assessments. Ongoing community engagement supports opportunities for future community-based intervention work.

The rapid response of SHOW investigators and longstanding partnerships with state health agencies to advance the COVID-19 response in Wisconsin and beyond, demonstrates the importance for maintaining such population health resources to address pressing public health priorities at a state and national level. When the pandemic began in early 2020, SHOW mobilized a series of three waves of longitudinal follow-up using online surveys and antibody surveillance to track impacts of COVID-19 over time within and across this study cohort. The study was facilitated by strong community-academic partnerships, ongoing relationships, and the unique expertise of the SHOW program staff in designing and supporting community-based sample collection. Thus, SHOW embodies all elements necessary to support population health sciences in the 21st century.

Unique strengths of the program include its well-designed geographically diverse study population, high quality and variable measures of social determinants of health, and carefully designed biorespository. Rigorous sampling strategies, and recruitment methods are employed to gather a breadth of data (over 2,000 variables). Geographic identifiers allow for linkage with community census and other social or environmental data. The biological samples collected from a non-clinical a non-clinical study sample are critical for advancing translational research from bench or clinic to community. This is particularly true for analysis of environmental exposure, and response to advance multi-omic and exposomic projects. The potential for long-term follow-up also enables new investigations of biological mechanisms of aging and health disparities across the life-course. With an average cohort age of 44 at baseline, the SHOW sample includes a significant number of genetically related (parent-child; siblings) and unrelated (husband-wife) participants with similar exposures or lifestyles. This sample structure allows unique opportunities to study genes, environment and family dynamics across the life-course.

New efforts in data integration, and method validation are also possible. With participating consent for use of data for future unspecified research and linkages with administrative data, numerous opportunities to expand core data. Ongoing research includes linkages with vital statistics, state cancer registry, and existing community level data. Increasingly new models of research are looking toward electronic health records for understanding health trajectories over time.

Despite significant strengths of the program, it is not without limitations. Conducting SHOW as a comprehensive population-based survey is both resource- and time-intensive. SHOW's sampling strategy was designed to ensure a statewide representative sample leading to both logistical and monetary costs. Although the resulting sample characteristics may be a strength for many types of epidemiological studies, it may be a limitation for other studies requiring a more substantial proportion of non-white participants, as the vast majority of state residents are white and <12% of the state's total population self-identifies as non-white. SHOW has recognized this limitation and in 2018–2019 conducted additional recruitment in more racially and ethnically diverse urban communities. Working in collaboration with communities requires long standing partnerships, trusted relationships and new approaches to sampling design and recruitment. These differences make some analyses of statewide data difficult. At the same time, working directly with communities offers new opportunities to for understanding data trends and for effectively promoting health and wellbeing.

## Data Availability Statement

Data are available for qualified investigators upon request, including the analytic files used to generate data in this manuscript. A fee for service model may apply for access to restricted data beyond that which is currently publicly available at www.show.wisc.edu.

## Ethics Statement

The studies involving human participants were reviewed and approved by the University of Wisconsin Health Services Institutional Review Board. Written informed consent to participate in this study was provided by all adult participants. For all minor participants, legal guardians provided written informed consent. Assent from the minors was also gathered when possible.

## Author Contributions

KM is the program Principal Investigator and is accountable for all aspects of the work and will ensure that all questions related to the accuracy or integrity of any part of the work are appropriately investigated and resolved. MN, TL, and AAS were involved in initial drafts of the manuscript. AR and LM supported edits for clarity and content accuracy. KM, TL, MN, AB, CE, and FN were responsible for drafting this manuscript or revising it critically for important content. All authors contributed to the planning, conduct of the SHOW cohort including contributions to the design, and acquisition or analysis of the work.

## Funding

This work was supported by the Wisconsin Partnership Program PERC Award [233 PRJ 25DJ and WPP4444], the National Institutes of Health's Clinical and Translational Science Award [5UL RR025011], and the National Heart Lung and Blood Institute [1 RC2 HL101468]. Ongoing ancillary study funding from the National Institutes of Health Include [X01HG010110], [R21AI142481] and [R01AG061080].Faculty supporting this research are also members of the Center for Demography and Ecology at the University of Wisconsin-Madison (P2C HD047873 and T32 HD07014) supported by a Eunice Kennedy Shriver National Institute of Child Health and Human Development grant to and the Center for Demography of Health and Aging at the University of Wisconsin-Madison supported by a National Institute on Aging grant (P30AG17266).

## Conflict of Interest

The authors declare that the research was conducted in the absence of any commercial or financial relationships that could be construed as a potential conflict of interest.

## Publisher's Note

All claims expressed in this article are solely those of the authors and do not necessarily represent those of their affiliated organizations, or those of the publisher, the editors and the reviewers. Any product that may be evaluated in this article, or claim that may be made by its manufacturer, is not guaranteed or endorsed by the publisher.

## References

[1] Nieto FJ Peppard PE Engelman CD McElroy JA Galvao LW Friedman EM . The Survey of the Health of Wisconsin (SHOW), a novel infrastructure for population health research: rationale and methods. BMC Public Health. (2010) 10:785. 10.1186/1471-2458-10-785 21182792 PMC3022857

[2] DeSalvo KB Wang YC Harris A Auerbach J Koo D O'Carroll P. Public health 3. 0: a call to action for public health to meet the challenges of the 21st century. Prev Chronic Dis. (2017) 14:E78. 10.5888/pcd14.170017 28880837 PMC5590510

[3] Burke LE Shiffman S Music E Styn MA Kriska A Smailagic A . Ecological momentary assessment in behavioral research: addressing technological and human participant challenges. J Med Internet Res. (2017) 19:e77. 10.2196/jmir.7138 28298264 PMC5371716

[4] Green-Harris G Coley SL Koscik RL Norris NC Houston SL Sager MA . Addressing disparities in Alzheimer's disease and African-American participation in research: an asset-based community development approach. Front Aging Neurosci. (2019) 11:125. 10.3389/fnagi.2019.00125 31214014 PMC6554429

[5] Schultz AA Malecki KMC Olson MM Selman SB Olaiya OI Spicer A . Investigating cumulative exposures among 3- to 4-year-old children using wearable ultrafine particle sensors and language environment devices: a pilot and feasibility study. Int J Environ Res Public Health. (2020) 17. 10.3390/ijerph17145259 32708240 PMC7400160

[6] Addissie YA Troia A Wong ZC Everson JL Kozel BA Muenke M . Identifying environmental risk factors and gene-environment interactions in holoprosencephaly. Birth Defects Res. (2020). 10.1002/bdr2.1834 33111505 PMC9288838

[7] Addissie YA Kruszka P Troia A Wong ZC Everson JL Kozel BA . Prenatal exposure to pesticides and risk for holoprosencephaly: a case-control study. Environ Health. (2020) 19:65. 10.1186/s12940-020-00611-z 32513280 PMC7278164

[8] Malecki KM Schultz AA Severtson DJ Anderson HA VanDerslice JA. Private-well stewardship among a general population based sample of private well-owners. Sci Total Environ. (2017) 601:1533–43. 10.1016/j.scitotenv.2017.05.284 28605871 PMC5662198

[9] Beyer KM Kaltenbach A Szabo A Bogar S Nieto FJ Malecki KM. Exposure to neighborhood green space and mental health: evidence from the survey of the health of Wisconsin. Int J Environ Res Public Health. (2014) 11:3453–72. 10.3390/ijerph110303453 24662966 PMC3987044

[10] Beyer KM Malecki KM Hoormann KA Szabo A Nattinger AB. Perceived neighborhood quality and cancer screening behavior: evidence from the survey of the health of Wisconsin. J Community Health. (2016) 41:134–7. 10.1007/s10900-015-0078-1 26275881 PMC4984667

[11] Givens ML Malecki KC Peppard PE Palta M Said A Engelman CD . Shiftwork, sleep habits, and metabolic disparities: results from the survey of the health of Wisconsin. Sleep Health. (2015) 1:115–20. 10.1016/j.sleh.2015.04.014 26894229 PMC4755509

[12] Guzman A Walsh MC Smith SS Malecki KC Nieto FJ. Evaluating effects of statewide smoking regulations on smoking behaviors among participants in the Survey of the Health of Wisconsin. WMJ. (2012) 111:166–71. 22970531 PMC3529004

[13] Laxy M Malecki KC Givens ML Walsh MC Nieto FJ. The association between neighborhood economic hardship, the retail food environment, fast food intake, and obesity: findings from the Survey of the Health of Wisconsin. BMC Public Health. (2015) 15:237. 10.1186/s12889-015-1576-x 25885908 PMC4409709

[14] Malecki KMC Schultz AA Bergmans RS. Neighborhood perceptions and cumulative impacts of low level chronic exposure to fine particular matter (PM2.5) on cardiopulmonary health. Int J Environ Res Public Health. (2018) 15. 10.3390/ijerph15010084 29316641 PMC5800183

[15] Saiz AM Jr Aul AM Malecki KM Bersch AJ Bergmans RS LeCaire TJ . Food insecurity and cardiovascular health: findings from a statewide population health survey in Wisconsin. Prev Med. (2016) 93:1–6. 10.1016/j.ypmed.2016.09.002 27612573 PMC6095702

[16] Schultz AA Schauer JJ Malecki KM. Allergic disease associations with regional and localized estimates of air pollution. Environ Res. (2017) 155:77–85. 10.1016/j.envres.2017.01.039 28193558 PMC6230689

[17] Shin JI Bautista LE Walsh MC Malecki KC Nieto FJ. Food insecurity and dyslipidemia in a representative population-based sample in the US. Prev Med. (2015) 77:186–90. 10.1016/j.ypmed.2015.05.009 26007296 PMC4608370

[18] VanWormer JJ Acharya A Greenlee RT Nieto FJ. Oral hygiene and cardiometabolic disease risk in the survey of the health of Wisconsin. Community Dent Oral Epidemiol. (2013) 41:374–84. 10.1111/cdoe.12015 23106415 PMC3566323

[19] Eggers S Remington PL Ryan K Nieto J Peppard P Malecki K. Obesity prevalence and health consequences: findings from the survey of the health of Wisconsin, 2008-2013. WMJ. (2016) 115:238–44. 29095585 PMC6230699

[20] Bailey EJ Malecki KC Engelman CD Walsh MC Bersch AJ Martinez-Donate AP . Predictors of discordance between perceived and objective neighborhood data. Ann Epidemiol. (2014) 24:214–21. 10.1016/j.annepidem.2013.12.007 24467991 PMC3947547

[21] Gorzelitz J Peppard PE Malecki K Gennuso K Nieto FJ Cadmus-Bertram L. Predictors of discordance in self-report versus device-measured physical activity measurement. Ann Epidemiol. (2018) 28:427–31. 10.1016/j.annepidem.2018.03.016 29681429 PMC6500726

[22] Malecki KC Engelman CD Peppard PE Nieto FJ Grabow ML Bernardinello M . The Wisconsin Assessment of the Social and Built Environment (WASABE): a multi-dimensional objective audit instrument for examining neighborhood effects on health. BMC Public Health. (2014) 14:1165. 10.1186/1471-2458-14-1165 25391283 PMC4289353

[23] Gorzelitz JS Malecki KM Cadmus-Bertram LA. Awareness of physical activity guidelines among rural women. Am J Prev Med. (2020) 59:143–5. 10.1016/j.amepre.2020.01.022 32564803 PMC7338004

[24] Hale L Hill TD Friedman E Nieto FJ Galvao LW Engelman CD . Perceived neighborhood quality, sleep quality, and health status: evidence from the Survey of the Health of Wisconsin. Soc Sci Med. (2013) 79:16–22. 10.1016/j.socscimed.2012.07.021 22901794 PMC3733364

[25] Johnson BS Malecki KM Peppard PE Beyer KMM. Exposure to neighborhood green space and sleep: evidence from the Survey of the Health of Wisconsin. Sleep Health. (2018) 4:413–9. 10.1016/j.sleh.2018.08.001 30241655 PMC6152838

[26] Martinez-Donate AP Riggall AJ Meinen AM Malecki K Escaron AL Hall B . Evaluation of a pilot healthy eating intervention in restaurants and food stores of a rural community: a randomized community trial. BMC Public Health. (2015) 15:136. 10.1186/s12889-015-1469-z 25885704 PMC4331304

[27] Malecki KMC Nikodemova M Schultz AA LeCaire TJ Bersch AJ Cadmus-Bertram L . The survey of the health of Wisconsin (SHOW) Program: an infrastructure for advancing population health sciences. medRxiv. (2021) 2021.03.15.21253478. 10.1101/2021.03.15.21253478 35433595 PMC9008403

[28] Said A Gagovic V Malecki K Givens ML Nieto FJ. Primary care practitioners survey of non-alcoholic fatty liver disease. Ann Hepatol. (2013) 12:758–65. 10.1016/S1665-2681(19)31317-1 24018493

[29] Denu RA Maloney J Tomasallo CD Jacobs NM Krebsbach JK Schmaling AL . Survey of radon testing and mitigation by Wisconsin residents, landlords, and school districts. WMJ. (2019) 118:169–76. 31978285 PMC7008351

[30] Cadmus-Bertram LA Gorzelitz JS Dorn DC Malecki KMC. Understanding the physical activity needs and interests of inactive and active rural women: a cross-sectional study of barriers, opportunities, and intervention preferences. J Behav Med. (2020) 43:638–47. 10.1007/s10865-019-00070-z 31197537 PMC7891881

[31] Litzelman K Skinner HG Gangnon RE Nieto FJ Malecki K Witt WP. Role of global stress in the health-related quality of life of caregivers: evidence from the Survey of the Health of Wisconsin. Qual Life Res. (2014) 23:1569–78. 10.1007/s11136-013-0598-z 24322907 PMC4032607

[32] Litzelman K Skinner HG Gangnon RE Nieto FJ Malecki K Witt WP. The relationship among caregiving characteristics, caregiver strain, and health-related quality of life: evidence from the Survey of the Health of Wisconsin. Qual Life Res. (2015) 24:1397–406. 10.1007/s11136-014-0874-6 25427430 PMC4446250

[33] Litzelman K Witt WP Gangnon RE Nieto FJ Engelman CD Mailick MR . Association between informal caregiving and cellular aging in the survey of the health of wisconsin: the role of caregiving characteristics, stress, and strain. Am J Epidemiol. (2014) 179:1340–52. 10.1093/aje/kwu066 24780842 PMC4036217

[34] Nikodemova M Yee J Carney PR Bradfield CA Malecki KM. Transcriptional differences between smokers and non-smokers and variance by obesity as a risk factor for human sensitivity to environmental exposures. Environ Int. (2018) 113:249–58. 10.1016/j.envint.2018.02.016 29459183 PMC5866236

[35] Bhutani S Schoeller DA Walsh MC McWilliams C. Frequency of eating out at both fast-food and sit-down restaurants was associated with high body mass index in non-large metropolitan communities in midwest. Am J Health Promot. (2018) 32:75–83. 10.1177/0890117116660772 27574335 PMC5453830

[36] Escaron AL Martinez-Donate AP Riggall AJ Meinen A Hall B Nieto FJ . Developing and implementing “Waupaca Eating Smart”: a restaurant and supermarket intervention to promote healthy eating through changes in the food environment. Health Promot Pract. (2016) 17:265–77. 10.1177/1524839915612742 26546508

[37] Martinez-Donate AP Espino JV Meinen A Escaron AL Roubal A Nieto J . Neighborhood disparities in the restaurant food environment. WMJ. (2016) 115:251–8. 29095587 PMC6095698

[38] Malecki K Wisk LE Walsh M McWilliams C Eggers S Olson M. Oral health equity and unmet dental care needs in a population-based sample: findings from the Survey of the Health of Wisconsin. Am J Public Health. (2015) 105 Suppl 3:S466–74. 10.2105/AJPH.2014.302338 25905843 PMC4455504

[39] Christensen KY Thompson BA Werner M Malecki K Imm P Anderson HA. Levels of nutrients in relation to fish consumption among older male anglers in Wisconsin. Environ Res. (2015) 142:542–8. 10.1016/j.envres.2015.08.005 26296180 PMC5010083

[40] Christensen KY Thompson BA Werner M Malecki K Imm P Anderson HA. Levels of persistent contaminants in relation to fish consumption among older male anglers in Wisconsin. Int J Hyg Environ Health. (2016) 219:184–94. 10.1016/j.ijheh.2015.11.001 26614251 PMC6095701

[41] Services WDoH. Wisconsin Cancer Plan 2020-2030. Madison, WI: Univeersity of Wisconsin Carbonee Cancer Center, University of Wisconsin Madison. (2020).

[42] Engelman CD Bo R Zuelsdorff M Steltenpohl H Kirby T Nieto FJ. Epidemiologic study of the C-3 epimer of 25-hydroxyvitamin D(3) in a population-based sample. Clin Nutr. (2014) 33:421–5. 10.1016/j.clnu.2013.06.005 23831447 PMC3884039

[43] Brown HW Wise ME LeCaire TJ Braun EJ Drewry AM Buttigieg EM . Reasons behind preferences for community-based continence promotion. Female Pelvic Med Reconstr Surg. (2020) 26:425–30. 10.1097/SPV.0000000000000806 32217918 PMC7329600

[44] Brennan PF Ponto K Casper G Tredinnick R Broecker M. Virtualizing living and working spaces: Proof of concept for a biomedical space-replication methodology. J Biomed Inform. (2015) 57:53–61. 10.1016/j.jbi.2015.07.007 26173040

[45] Casper GR Brennan PF Arnott Smith C Werner NE He Y. Health@Home Moves All About the House! Stud Health Technol Inform. (2016) 225:173–7. 27332185 PMC5546307

[46] Casper GR Flatley Brennan P Perreault JO Marvin AG. vizHOME–A context-based home assessment: Preliminary implications for informatics. Stud Health Technol Inform. (2015) 216:842–6. 26262170

[47] Bautista LE Bajwa PK Shafer MM Malecki KMC McWilliams CA Palloni A. The relationship between chronic stress, hair cortisol and hypertension. Int J Cardiol Hypertens. (2019) 2. 10.1016/j.ijchy.2019.100012 33447745 PMC7803047

[48] Christensen K Werner M Malecki K. Serum selenium and lipid levels: associations observed in the national health and nutrition examination survey (NHANES) 2011–2012. Environ Res. (2015) 140:76–84. 10.1016/j.envres.2015.03.020 25836721

[49] Raymond MR Christensen KY Thompson BA Anderson HA. Associations between fish consumption and contaminant biomarkers with cardiovascular conditions among older male anglers in Wisconsin. J Occup Environ Med. (2016) 58:676–82. 10.1097/JOM.0000000000000757 27253229

[50] Christensen KY Raymond M Thompson BA Anderson HA. Perfluoroalkyl substances in older male anglers in Wisconsin. Environ Int. (2016) 91:312–8. 10.1016/j.envint.2016.03.012 27003842

[51] Knobeloch L Imm P Anderson H. Perfluoroalkyl chemicals in vacuum cleaner dust from 39 Wisconsin homes. Chemosphere. (2012) 88:779–83. 10.1016/j.chemosphere.2012.03.082 22542201

[52] Yu D Richardson NE Green CL Spicer AB Murphy ME Flores V . The adverse metabolic effects of branched-chain amino acids are mediated by isoleucine and valine. Cell Metab. (2021) 33:905–22 e6. 10.1016/j.cmet.2021.03.025 33887198 PMC8102360

[53] Eggers S Malecki KM Peppard P Mares J Shirley D Shukla SK . Wisconsin microbiome study, a cross-sectional investigation of dietary fibre, microbiome composition and antibiotic-resistant organisms: rationale and methods. BMJ Open. (2018) 8:e019450. 10.1136/bmjopen-2017-019450 29588324 PMC5875638

[54] Schultz AA Malecki KM Holzhausen EA Bajwa P Peppard P LeCaire T . The population-based microbiome research core: a longitudinal infrastructure for assessment of household microbiome and human health research. medRxiv. (2021) 2021.11.22.21266369. 10.1101/2021.11.22.21266369

[55] Eggers S Safdar N Sethi AK Suen G Peppard PE Kates AE . Urinary lead concentration and composition of the adult gut microbiota in a cross-sectional population-based sample. Environ Int. (2019) 133:105122. 10.1016/j.envint.2019.105122 31518933 PMC7230144

[56] Kates AE Jarrett O Skarlupka JH Sethi A Duster M Watson L . Household pet ownership and the microbial diversity of the human gut microbiota. Front Cell Infect Microbiol. (2020) 10:73. 10.3389/fcimb.2020.00073 32185142 PMC7058978

[57] Malecki KMC Schultz AA Nikodemova M Walsh MC Bersch AJ Cronin J . Statewide impact of COVID-19 on social determinants of health - a first look: findings from the survey of the health of Wisconsin. medRxiv. (2021) 2021.02.18.21252017. 10.1101/2021.02.18.21252017

[58] Malecki K Nikodemova M Schultz A Walsh M Bersch A Sethi A . Population changes in seroprevalence among a statewide sample in the United States. medRxiv. (2020) 2020.12.18.20248479. 10.1101/2020.12.18.20248479

