# Supplementary material for: The Survey of the Health of Wisconsin (SHOW) Program: An Infrastructure for Advancing Population Health
Source: Front Public Health. 2022 Mar 31;10:818777. doi: 10.3389/fpubh.2022.818777 (PMC9008403; doi:10.3389/fpubh.2022.818777)
Supplement: Supplementary file 1 [file Data_Sheet_1.docx]

**Supplemental Table 1A. SHOW Participation by Health Region or County by Waves I - III, 2008-2017.**

| **Health Region** | **WAVE I**  **2008-2013** | **County in Health Region** | **WAVE II**  **2014-2016** | **WAVE III**  **2017** |
| --- | --- | --- | --- | --- |
| North | 66.5% | Wood | 60.1% | 100% |
| Northeast | 57.8% | Brown | 64.7% | 82.3% |
|  |  | Waushara | 80.2% |  |
| South | 62.0% | Dane | 65.3% | 88.8% |
| Southeast | 50.8% | Milwaukee | 54.4% | 85.3% |
|  |  | Racine | 63.1% |  |
|  |  | Ozaukee | 63.0% |  |
| West | 59.9% | La Crosse | 53.0% | 80.0% |
|  |  | Eau Claire | 71.7% |  |
|  |  | Polk | 73.1% |  |

**Supplemental Table 1B. SHOW Participation Rates by WAVE and Urbanicity.** Rates are estimated as the percent of adult individuals who screened eligible who agree to participate based on cohort year and urban/rural status of resident census tract. A more detailed summary of participation rates by health region (2008-2013 and 2017) and by County (2014-2016) is presented in Supplemental Table 1 and available online.

| Response Rates | Overall  % | Urban  % | Rural  % |
| --- | --- | --- | --- |
| WAVE I | 57.5 | 56.1 | 60.5 |
| WAVE II | 63.5 | 62.0 | 70.4 |
| WAVE III | 85.6 | 84.9 | 85.9 |

|  | **WAVE I**  **SHOW 2008-2013**  21-74 years old | **WAVE II**  **SHOW 2014-2016**  All ages | **WAVE III***  **SHOW 2017 follow up**  All ages | **WAVE IV** |
| --- | --- | --- | --- | --- |
| **Questionnaires** |  |  |  |  |
| *Demographics* | ✓ | ✓ | ✓ | ✓ |
| *Health and health history* | ✓ | ✓ | ✓ | ✓ |
| *Mental health* | ✓ | ✓ | ✓ | ✓ |
| *Health care and medication* | ✓ | ✓ | ✓ | ✓ |
| *Health related behaviors* | ✓ | ✓ | ✓ | ✓ |
| *Physical and built environment* | ✓ | ✓ | ✓ | ✓ |
| *Social and economic determinants* | ✓ | ✓ | ✓ | ✓ |
| **Clinical measurements** |  |  |  |  |
| *Weight* | ✓ | ≥ 3 years old | ≥ 3 years old | ≥ 3 years old |
| *Height* | ✓ | ≥ 3 years old | ≥ 3 years old | ≥ 3 years old |
| *Waist and hip circumference* | ✓ | ≥ 3 years old | ≥ 3 years old | ≥ 3 years old |
| *Bioimpedance* | ✓ |  |  |  |
| *Blood pressure and heart rate* | ✓ | ≥ 3 years old | ≥ 3 years old | ≥ 3 years old |
| *Spirometry (lung function)* | ✓ | ≥ 6 years old | ≥ 6 years old | ≥ 6 years old |
| **Accelerometry (hip, wrist)** |  | ≥ 6 years old | ≥ 6 years old | ≥ 6 years old |
| **Blood testing** |  |  |  |  |
| *CBC* | ✓ | ≥ 18 years old | ≥ 18 years old | ≥ 18 years old |
| *Triglycerides* |  | ≥ 18 years old | ≥ 18 years old | ≥ 18 years old |
| *Total and HDL cholesterol* | ✓ | ≥ 18 years old | ≥ 18 years old | ≥ 18 years old |
| *HbA1c* | ✓ | ≥ 18 years old | ≥ 18 years old | ≥ 18 years old |
| *Glucose* | ✓ | ≥ 18 years old | ≥ 18 years old | ≥ 18 years old |
| *Creatinine* | ✓ | ≥ 18 years old | ≥ 18 years old | ≥ 18 years old |
| **Biosample collection and banking** |  |  |  |  |
| *Serum* | ✓ | ≥ 18 years old | ≥ 18 years old | ≥ 18 years old |
| *Plasma* | ✓ | ≥ 18 years old | ≥ 18 years old | ≥ 18 years old |
| *Urine* | ✓ | ≥ 18 years old | ≥ 18 years old | ≥ 18 years old |
| *DNA* | ✓ | ≥ 18 years old | ≥ 18 years old | ≥ 18 years old |
| *PAXgene tubes for RNA* |  | ≥ 18 years old | ≥ 18 years old | ≥ 18 years old |
| *Stool, nasal, skin swab* |  | ≥ 18 years old  only in 2016 | ≥ 18 years old  subset | ≥ 18 years old  subset |

**Supplemental Table 2: Survey components WAVES I-IV**

* Phase III was a follow- up survey of adults participating in SHOW Phase I during which children were not included. Children living in Phase I households in 2017 were eligible to participate in Phase III. Children enrolled in Phase III completed a baseline survey.
